# Spectroscopy and chromaticity characterization of yellow to light-blue iron-containing beryl

**DOI:** 10.1038/s41598-022-11916-z

**Published:** 2022-06-24

**Authors:** Yanran Shang, Ying Guo, Jun Tang

**Affiliations:** School of Gemmology, China University of Geosciences (Beijing), No.29 Xueyuan Road, Beijing, 100083 China

**Keywords:** Materials science, Optics and photonics, Physics

## Abstract

The chemical composition and influencing factors of the colour of 95 yellow to light blue iron-bearing beryl are studied through Electron Microprobe Analysis (EMPA), Energy-dispersive X-ray fluorescence (ED-XRF) spectroscopy, Fourier transform infrared (FTIR) spectroscopy, ultraviolet–visible (UV–vis) spectroscopy and X-Rite SP62 spectrophotometer. The intensities of the three characteristic hydroxyl stretching bands of the beryl from 3500 cm^−1^ to 3800 cm^−1^ prove they are low to medium levels of alkali bearing natural beryl. The wide absorption edge of 320 ~ 465 nm caused by the ultra-violet charge transfer from O^2−^ to Fe^3+^ and the 650 nm absorption band in E//c-polarization caused by the intervalence charge transfer between Fe^2+^ and Fe^3+^ are the main factors affecting the colour of beryl. By applying CIE D_65_ standard light source and N9 Munsell neutral background as testing conditions, the colour parameters of 82 gem-quality beryl are tested. According to the results, iron-containing beryl colours are classified into yellow, yellowish-green, bluish-green, greenish-blue, and blue by the K-means cluster analysis method. The blue tone has a greater influence on the hue of beryl, while the yellow tone has a greater influence on the chroma. Iron content is higher in yellow and blue beryl. With the increase of iron content, the lightness of beryl decreased and the chroma increased.

## Introduction

Beryl is a kind of hexagonal cyclosilicate (Be_3_Al_2_Si_6_O_18_). The [SiO_4_]^4−^ tetrahedron forms a six-membered ring paralleling to the (0001) plane, and the Si rings are connected by twisted [BeO_4_]^6−^ tetrahedron and [AlO_6_]^9−^ octahedron. The center of the six-membered ring forms an open channel paralleling to the c axis, which accommodates alkalis, transition metal cations and large molecules such as water and carbon dioxide^1,2^. There are two kinds of free water in beryl channel: type-I and type-II with different orientations. The orienting forces on the type-I water are due to remote electrostatic charge which leads the symmetry axis perpendicular to the c axis^3^**.** Type-I water rotates from parallel direction to vertical direction under the electric field of alkali located at the 2b position, and it turns into type-II water^4,5^.

In the crystal structure of beryl, the occurrence of ion substitution is very common, resulting in a variety of complex colours. Colourless beryl with no impurity is called goshenite ^6^. Emerald is coloured by Cr or V^7^. When Mn^3+^ replaces Al^3+^, morganite is formed^8^. The wide range of iron-containing beryl includes several subspecies such as heliodor, golden beryl^9,10^, aquamarine^11^, and Maxixe beryl^12,13^.

Most of the researches about iron-containing beryl are mainly focused on crystal physics and chemistry. However, there are few studies focusing on the colour and appearance of beryl. In recent years, colourimetry has played an important role in mineral colour quantification and classification. It is widely used in gemmology to find suitable light source^14–16^and background for gemstones^17,18^, for some colour-changing gemstones, different illumination may even completely affect the appearance of colour^19,20^. It was introduced to study the colour mechanism of gemstone, particularly turquoise^21^, chrysoprase ^22^, amethyst ^23^ and Xiuyan jade^24^.

Modern colourimetry is mainly established in the CIE 1976 L*a*b* colour system, which is based on human physiological characteristics, and describes human visual induction digitally, so it is closer to the effect seen by human eyes. The L*a*b* colour system is composed of three parameters, L* represents lightness whose value varies between 0 and 100 showing the gradual change from black to white, while a* and b* parameters are used to record the colour information. a* indicates the transition from green to red, the saturation of red increases with the increase of positive axis value, and the saturation of green is proportional to the absolute value of negative axis. 0 stands for the neutral gray. b* represents a blue-yellow transition. Parameters a* and b* jointly determine chroma *C*_*ab*_ and hue *h*_*ab*_, which reflect colour characteristics more intuitively. The calculations are as follows:

1 $$C_{ab} = \sqrt {a^{2} + b^{2} }$$

2 $$h_{ab}^{^\circ } = \tan^{ - 1} b/a$$

In this work, Electron Microprobe Analysis, Energy-dispersive X-ray fluorescence, Fourier transform infrared spectroscopy, ultraviolet–visible spectroscopy and colourimetry methods were applied to study the chemical composition and the colouration mechanism of yellow to light-blue iron-containing beryl.

## Results and discussion

### Chemical composition

Each sample was tested at one point because of the high clarity and the uniformity of the composition of the crystal. The result shows that the main elements of beryl crystal are Si (up to 63.18 SiO_2_ wt.%) and Al (up to 18.50 Al_2_O_3_ wt.%). The trace element with the highest content is Fe (up to 0.75 FeO_tot_ wt.%), and other minor contents are ≤ 0.62 wt.% Na_2_O, ≤ 0.22wt.% Nd_2_O_3_, ≤ 0.10 wt.% MgO, ≤0.09 wt.% TiO_2_, ≤ 0.08 wt.% K_2_O, ≤0.06 wt.% Cr_2_O_3_ and ≤0.06 wt.% F.

**Table 1:** The crystal chemical formula of beryl.

Sample number	Theoretical crystal chemical formula
Icb.44	Be_2.90~2.94_Al_2.06_Si_5.95~5.97_O_18_
Icb.46	Be_2.91~2.94_Al_2.07~2.08_Si_5.94~5.96_O_18_
Icb.48	Be_2.91~2.94_Al_2.07~2.08_Si_5.94~5.95_O_18_
Icb.50	Be_2.92~2.94_Al_2.06_Si_5.95~5.97_O_18_
Icb.51	Be_2.91~2.94_Al_2.06~2.07_Si_5.94~5.96_O_18_
Icb.52	Be_2.91~2.93_Al_2.08~2.09_Si_5.94~5.95_O_18_
Icb.53	Be_2.90~2.93_Al_2.08_Si_5.94~5.95_O_18_
Icb.54	Be_2.91~2.94_Al_2.05~2.06_Si_5.95~5.97_O_18_

Due to the limitation of EMPA in the field of ultra-light element analysis, H, Li and Be in beryl cannot be measured directly. However, the theoretical crystal chemical formula of beryl can be obtained by using ideal chemical ratio method.

The ideal crystal chemical formula of beryl is Be_3_Al_2_Si_6_O_18_. Therefore, it is assumed that the total number of cations in the sample is 11 and the total number of positive charges is 36. Taking the data of Icb.46 as an example, the concrete steps of the ideal chemical ratio method are introduced.

The cationic coefficients of Si, Al, Fe, Cr and Mg are calculated. Because the valence state of iron is unknown, the crystal chemical formulas are calculated by regarding total iron as Fe^2+^ or Fe^3+^ respectively:

Assuming that total iron is Fe^2+^, there are the following relationships:

3 $$\left( {{\mathrm{X}}_{{{\mathrm{Be}}}} + {\mathrm{X}}_{{{\mathrm{Si}}}} + {\mathrm{X}}_{{{\mathrm{Al}}}} + {\mathrm{X}}_{{{\mathrm{Mg}}}} + {\mathrm{X}}_{{{\mathrm{Cr}}}} + {\mathrm{X}}_{{{\mathrm{Fe}}}}^{{{2} + }} } \right) \cdot {\mathrm{X}} = {11}$$

4 $$\left( {{\mathrm{2X}}_{{{\mathrm{Be}}}} + {\mathrm{4X}}_{{{\mathrm{Si}}}} + {\mathrm{3X}}_{{{\mathrm{Al}}}} + {\mathrm{2X}}_{{{\mathrm{Mg}}}} + {\mathrm{3X}}_{{{\mathrm{Cr}}}} + {\mathrm{2X}}_{{{\mathrm{Fe}}}}^{{{2} + }} } \right) \cdot {\mathrm{X}} = {36}$$

Assuming that total iron is Fe^3+^, the relationships are as follows:

5 $$\left( {{\mathrm{X}}_{{{\mathrm{Be}}}} + {\mathrm{X}}_{{{\mathrm{Si}}}} + {\mathrm{X}}_{{{\mathrm{Al}}}} + {\mathrm{X}}_{{{\mathrm{Mg}}}} + {\mathrm{X}}_{{{\mathrm{Cr}}}} + {\mathrm{X}}_{{{\mathrm{Fe}}}}^{{{3} + }} } \right) \cdot {\mathrm{X}} = {11}$$

6 $$\left( {{\mathrm{2X}}_{{{\mathrm{Be}}}} + {\mathrm{4X}}_{{{\mathrm{Si}}}} + {\mathrm{3X}}_{{{\mathrm{Al}}}} + {\mathrm{2X}}_{{{\mathrm{Mg}}}} + {\mathrm{3X}}_{{{\mathrm{Cr}}}} + {\mathrm{3X}}_{{{\mathrm{Fe}}}}^{{{3} + }} } \right) \cdot {\mathrm{X}} = {36}$$

By solving the above equations, the result shows that X_Be_ = 0. 5079, X = 5. 7265, so the coefficient of Be is 2.9088 when iron ion is divalent. X_Be_ = 0. 5139, X = 5. 7109 and the coefficient of Be is 2.9351 when the iron ion is trivalent. When Fe^2+^ and Fe^3+^ exist together in beryl, the coefficient of Be in crystal chemical formula should be 2.9088 ~ 2.9351.

The chemical formula of Icb.46 is Be_2.91~2.94_(Al_2.07~2.08_Fe_0.04_)Si_5.96_O_18_(0.01Na,0.01 K). Trace elements are generally not written into the crystal chemical formula of beryl, so the crystal chemical formula is abbreviated as Be_2.91~2.94_Al_2.07~2.08_Si_5.96_O_18_, which is very similar to the formula calculated by Taran^25^.

The crystal chemical formulas of the remaining seven samples were calculated in the same way, and the results are shown in Table 1. According to Table 1, the chemical formula of the tested samples is Be_2.90 ~ 2.94_ Al_2.05 ~ 2.09_ Si_5.94 ~ 5.97_ O_18_.

XRF data are used to analyze the relationship between iron content and beryl colour parameters, which will be discussed in the later paragraph.

### Infrared spectral characteristics

**Figure 1:**
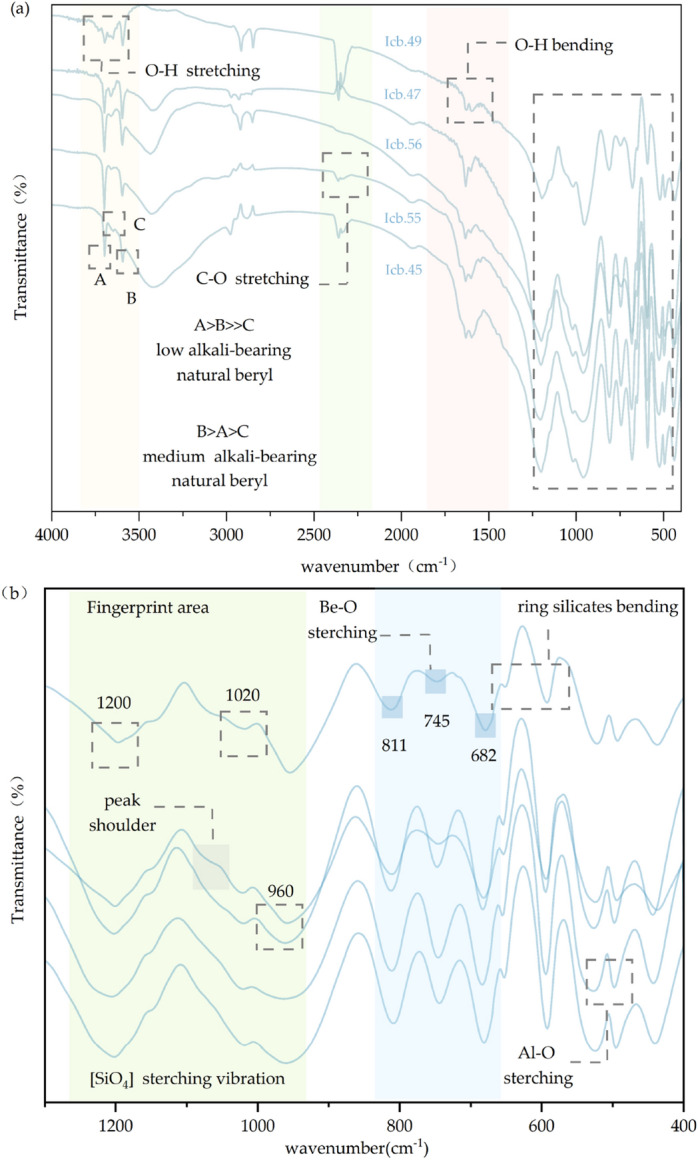
FTIR spectra of beryl. (**a**) 4000 cm^−1^ ~ 1300 cm^−1^ indicates samples are beryl with low to medium alkali content, the existence of carbon dioxide absorption peak proves that the samples are natural beryl. (**b**) Fingerprint region of infrared spectrum, which shows the characteristic absorptions of beryl. The spectra are displaced vertically for clarity.

The FTIR spectra of five beryls are given in Fig. 1. Two different kinds of water in channel show different absorption bands. Type-I water has stretching vibrations at 3698 cm^−1^ and bending vibration at 1600 cm^−1^. The stretching vibrations of type- II water with the bands at 3663 cm^−1^ and 3600 cm^−1^, as well as bending vibration at 1635 cm^−1^ are related with alkali ^26^.

The absorption increases in intensity as the alkali content of the beryl rises^27^, therefore the three O–H stretching bands of 3800–3500 cm^−1^ can help us quickly identify the types of beryl^28,29^. According to Schmetzer and Kiefert^30^, the strong absorption band at 3694 cm^−1^ is designated band A. The second absorption band at 3592 cm^−1^ is designated band B. The absorption between bands A and B, at 3655 cm^−1^ is called band C. The positions of these three bands are slightly offset in the infrared spectrum of my beryl samples, their positions have been marked in Fig. 1a. The absorption intensity of these three bands of low-alkali bearing beryl is A > B > > C (Transmittance is inversely proportional to absorption intensity), and of medium-alkali bearing beryl is B > A > C. The result shows that Icb.45, Icb.47 and Icb.49 have a medium alkali content in the structural channel. However, in Icb55 and Icb.56, there are few alkali ions.

The infrared absorption bands in the range of 1300 cm^−1^ ~ 400 cm^−1^ called fingerprint area reflect the stretching and bending vibration of beryl structure^31,32^, shown in Fig. 1b. The absorptions at 1196 cm^−1^,1020 cm^−1^ and 955 cm^−1^ are related to the stretching vibrations of Si–O in *E*_*1u*_^33^. The absorption band at 1196 cm^-1^ shifts to a higher wavenumber 1200 cm^-1^. The absorption band at 1020 cm^−1^ increases in intensity and broadens with extent of substitution in the octahedrally site, conversely it shrinks with the increasing extent of tetrahedral substitution. The curve of this absorption band is narrow and low, and it conforms to Aurisicchio's "normal" beryl^34^. The small absorptions shoulder of 1070 cm^−1^ are absent in Icb.45, Icb.55 and Icb.56, indicating the tetrahedral substitutions are not very common in the samples.

Absorption bands around 811 cm^−1^, 745 cm^−1^ and 682 cm^−1^ are mostly related to stretching vibrations of Be–O, and absorption bands at 650 and 590 cm^−1^ are characteristic bands for ring silicates^35^. Bands of Al–O vibrations is around 525 and 495 cm^−1^^36^. The band shift increases with the increase of octahedral distortion, especially when Al is replaced by larger divalent cations. These two bands shift slightly to low wavenumber, indicating that there are few ion substitutions et al. site^34^.

According to the 811 nm absorption position, it could be inferred that the degree of tetrahedral substitution is less than 4%. Similarly, the shift at 1196 nm indicates that the octahedral substitution of our sample is less than 5%^35^. FTIR results show that there are few tetrahedral and octahedral substitutions, so this might be the explanation for the low chroma of our beryl colour.

The weak absorption band at 2360 cm^−1^correlates to the asymmetric stretching mode of CO_2_ vibration which is commonly present in natural materials, but is absent in synthetic ones^37,38^ .

### Colouration mechanism

The yellow colour of beryl comes from absorption edge between 320 and 465nm^39^. Wood and Nassau attributed the yellow colour to the ultra-violet charge transfer between Fe^3+^ substituted for octahedral Al^3+^ position and surrounding oxygen^3^. While Goldman and Rossman^40^ believed that the Fe^3+^ in the channel is responsible for the golden-yellow colour. Spinolo et al.^41^ described that this absorption is caused by the colour center. Platonov et al.^42^divided the yellow beryl into heliodor and golden beryl. Heliodor refers to beryl with Fe^3+^ replacing Al^3+^, while golden beryl originates from the substitution of Fe^3+^ for Be^2+^. Andersson^43^ gave a simple electron trap model: the electron donor is Fe^3+^ at tetrahedral or octahedral position. When the crystal is heated, electrons are released from the trap and Fe^3+^ is converted into Fe^2+^.

**Figure 2:**
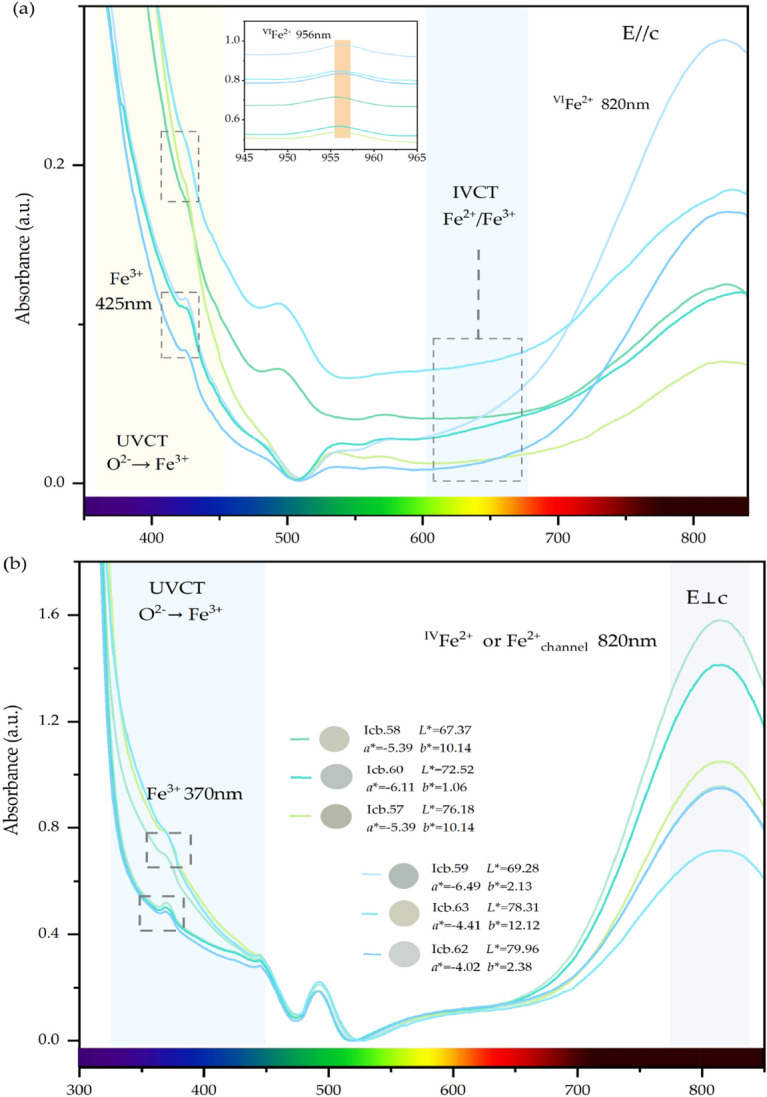
The polarized UV–vis spectra of beryl. (**a**) The E//c-polarization spectrum (**b**) The E⊥c-polarization spectrum.

The polarized UV–vis spectra of the sample are shown in Fig. 2. The absorption edge of purple-blue area leads to the yellow colour of beryl. There is no obvious difference between E//c and E⊥c. Absorption edges exist in all beryl samples, but the absorption edges of non-yellow beryl samples are in ultraviolet region. When this absorption edge shifts from violet-blue to longer wavelengths into the blue region, yellow appears in beryl. The spectrum shows the presence of ultraviolet charge transfer below 400 nm, it shifted to the blue region, so the beryl forms a yellow appearance^44^.

The band at 620 nm in polarization E//c absorbs red light and makes beryl blue. It was firstly assigned to the Fe^2+^ ions in the channel ^3^ and was later considered to be intervalence charge transfer between Fe^2+^ substituting Al^3+^ and Fe^3+^ located at 6 g position^45,46^.

The absorptions at 370 nm and 425 nm correspond to the spin-forbidden transitions of Fe^3+^ (^6^A_1g_ → ^4^T_2g_ and ^6^A_1g_ → ^4^E_g_ + ^4^A_1g_) in the octahedral site^47^. In the blue-tone beryl samples, these two absorptions are always present, while in the yellow-tone samples, this region is invisible covered by the absorption edge of the ultraviolet-blue region^48^. These two absorptions are weak and close to the ultraviolet region, so they make no contribution to beryl colour.

The strong absorption peak at 820 nm may originate from tetrahedral, octahedral or channel divalent iron^49^. It is a characteristic band of the UV–vis spectrum of beryl. In different polarization directions, this absorption has different origins. In E//c-polarization, it is attributed to Fe^2+^ substituting for Al^3+^ in octahedral sites. While in E⊥c-polarization, this position may indicate that the Fe^2+^ in the tetrahedral site or the channel site.

The band at 956 nm only appears in the E//c-polarized UV–Vis spectra is attributed to spin-allowed ^5^T_2g_ → ^5^E_g_ transitions of ^VI^Fe^2+^^50^. It has the same origin as the absorption at 820 nm. Although these two absorbances are located outside the visible light region and have no contribution to colour, they both reveal the existence of divalent iron.

### Colour characteristics and classification

Applying CIE D_65_ light source and N9 Munsell neutral background as testing conditions, the colour parameters of 82 gem-quality beryl are tested. Hue and chroma are calculated by a* and b*. The experimental result shows that the lightness range of beryl (63.37, 90.80), colourimetric coordinates a* (− 12.34, -3.55) and b* (-11.70, 25.26), chroma C* (4.32, 25.76) and hue *h°* (99.46, 223.48).

Pearson correlation coefficient “r” is used to reflect the linear correlation degree of two variables X and Y, and the value of r is between -1 and 1. The larger the absolute value, the stronger the correlation. when |*r*|< 0.4, there is weak or no correlation; when 0.4 ≤|*r*|< 0.6, there is moderate correlation; when 0.6 ≤|*r*|< 0.8, there is high correlation; when |*r*|≥ 0.8, there is extremely high correlation.

**Figure 3:**
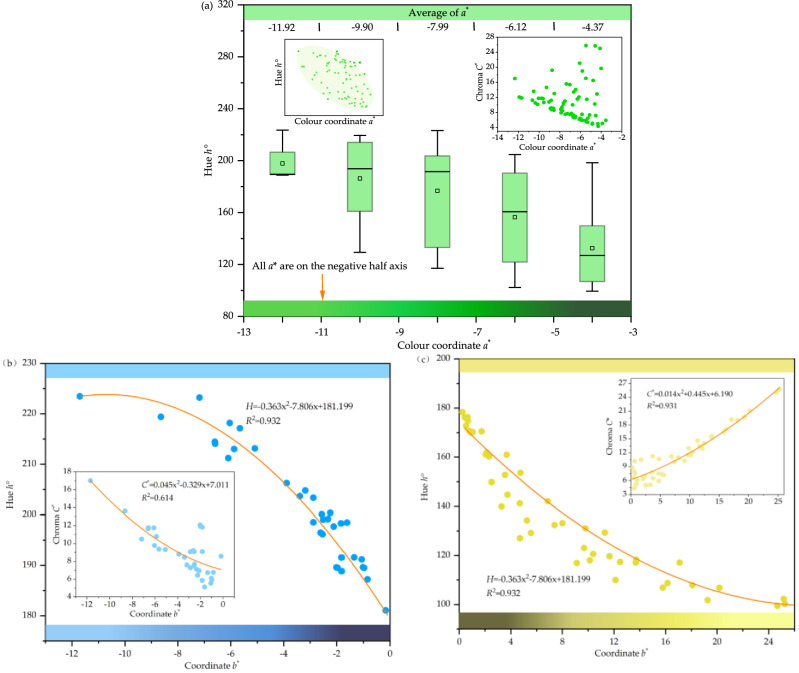
The colour analysis of beryl. (**a**) A negative correlation between the colour coordinate a* and its chroma C*. (**b**) Chromaticity coordinate − b* is highly negatively correlated with hue *h*°, and the colour coordinate -b* is high negative correlated with chroma C* (**c**) Chromaticity coordinate + b* is highly positively correlated with chromaticity C* and highly negatively correlated with hue *h*°.

By analyzing the colour data of 82 beryl, it is found that the colour coordinate a* is approximately negatively correlated with its Hue *h°*, as shown in Fig. 3a. The absence of the positive half axis of a* indicates that the colour of beryl is not controlled by red but by green. -b* shows the blue tone and + b* represents the yellow tone of the sample. Figure 3b and Fig. 3c shows there are 34 blue tone samples and 48 yellow -tone samples. the colour coordinate − b* is extremely high negative correlated with hue *h*° (Pearson's *r* = − 0.927, *R*^2^ = 0.932), and + b* is extremely high negative correlated with hue *h*° (Pearson's *r* = 0.908, *R*^2^ = 0.932).

The colour coordinate + b* is extremely high positive correlated with its chroma C* The colour coordinate + b* is extremely high positive correlated with its chroma C* (Pearson's *r* = 0.956, *R*^2^ = 0.931), and the colour coordinate − b* is high negative correlated with its chroma C* (Pearson's *r* = − 0.770, *R*^2^ = 0.614), indicating that the chroma of beryl is controlled by both blue and yellow tones, the influence degree of yellow is much stronger.

Compared with a*, the correlation between b* and hue *h*° is stronger, so the hue and chroma of beryl are mainly controlled by b*. The blue colour has a greater influence on the hue angle of beryl, while the yellow colour has a greater influence on the chroma.

The result of the colour tests shows that the hue angle of beryl ranges from 99 to 225, with an average *h°* of 164. The hue angle difference is about 126, which proves that the beryl samples we used in the experiment have a wide range of colour, and the experimental results are universal and representative. Because of the rich colour of iron-containing beryl, it is necessary for subsequent discussion to classify its colour. K-means cluster analysis is carried out with three independent colour parameters, L*, a* and b*. 3, 5, 7 and 9 are tried in turn to find the best classification scheme by comparing the number of cases and significant differences among groups. It is found that when the number of clusters is 5, the clustering effect is the best (Sig. < 0.001). The variance analysis results obtained are shown in Table 2.

**Table 2:** Cluster variance analysis of beryl colour.

Colour parameter	Sum of Squares	df	Mean Square	df	F	Sig
L*	378.179	4	8.522	77	44.377	.000
a*	1159.222	4	9.976	77	116.196	.000
b*	32.148	4	2.736	77	11.749	.000

Fisher discriminant function is used to test the clustering effects, the colour discriminant functions corresponding to five types of beryl are obtained as follows: The colour parameters L*, a*, b* are substituted back into five Fisher discriminant functions to discriminate, and the correct rate is 97.6%, which is in accordance with the ideal accuracy, indicating that the beryl colour classification scheme is effective.

7 $${\mathrm{F}}2 = 9.009L^{*} - 6.185a^{*} + 5.096b^{*} - 409.470$$

8 $${\mathrm{F}}2 = 9.216L^{*} - 6.677a^{*} + 3.923b^{*} - 408.843$$

9 $${\mathrm{F}}3 = 9.742L^{*} - 6.308a^{*} + 2.871b^{*} - 444.056$$

10 $${\mathrm{F}} 4 = 8.812L^{*} - 6.578a^{*} + 2.541b^{*} - 369.180$$

11 $${\mathrm{F}}5 = 8.373L^{*} - 7.329a^{*} + 1.909b^{*} - 344.104$$

**Figure 4:**
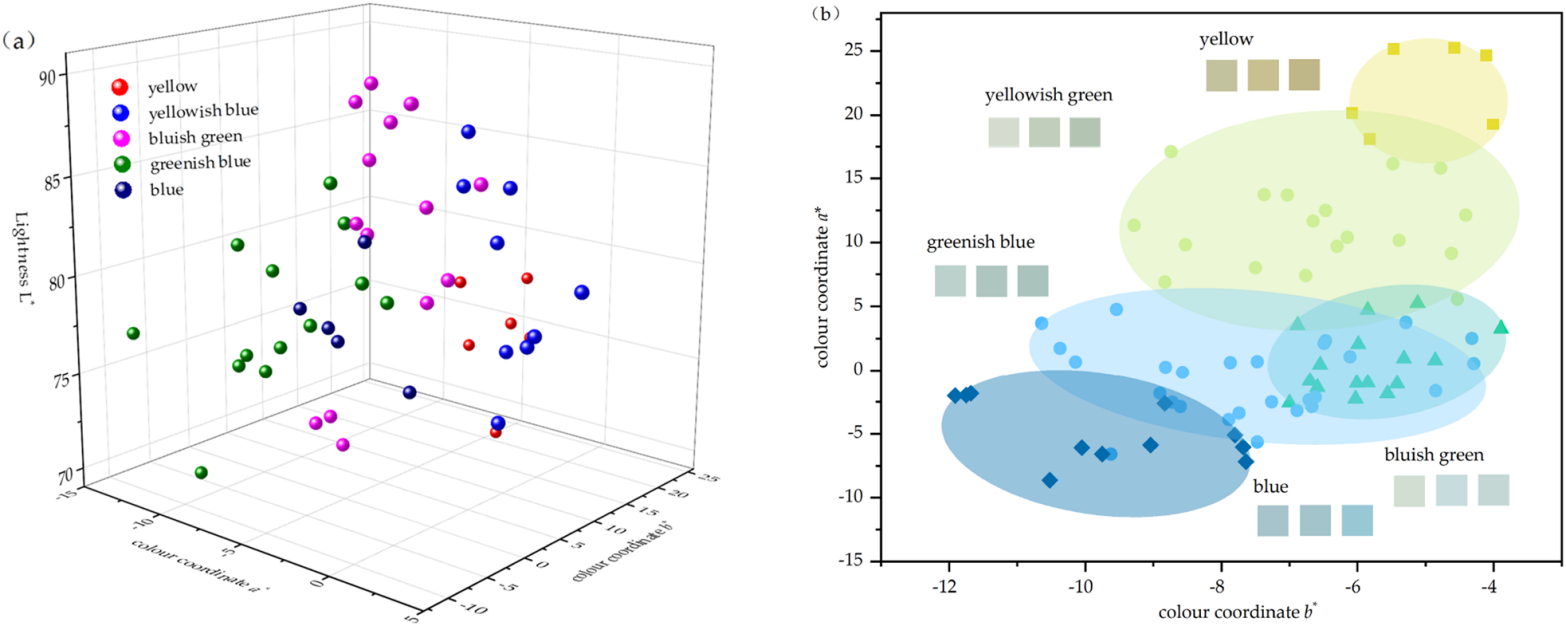
Colour classification of beryl applying CIE D_65_ standard light source and N9 Munsell background. (**a**) According to the result of K-means clustering, the colours of the 82 beryl samples can be divided into five groups. (**b**) The five groups of beryl colour: yellow, yellowish green, bluish green, greenish blue and blue.

According to the colour characteristics of each group, beryls are divided into yellow, yellowish green, bluish green, greenish blue and blue. The average values of the three parameters L*, C* and *h°* and the simulated colour block of the five groups of samples are shown in Table 3. The distribution of five groups in three-dimensional space is shown in Fig. 4a. Since the projection points in three-dimensional space are not intuitive enough, a chromaticity diagram is made in two-dimensional space with a* as X axis and b* as Y axis, as shown in Fig. 4b.

**Table 3:** The five average centres of K-Means clustering.

	Yellow	Yellowish green	Bluish green	Greenish blue	Blue
Number of samples	6	20	16	28	12
	74.6	78.91	86.88	78.01	74.38
	22.67	12.79	5.88	7.43	11.33
	102.77	121.22	174.43	183.32	208.87
Simulated colour

### Relationship between iron content and colour

**Figure 5:**
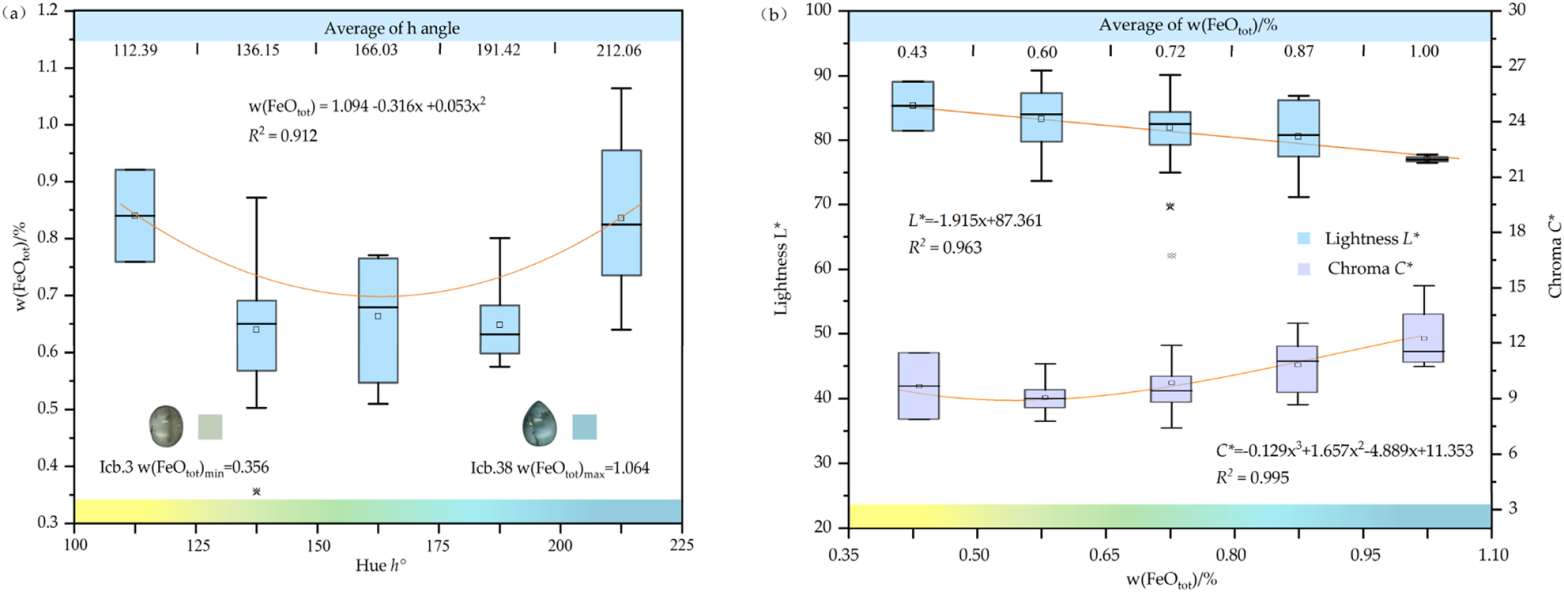
Relationship between iron and colour in beryl. (**a**) The iron content of beryl has a quadratic function with hue angle, and yellow and blue beryl have the highest iron content. (**b**) The iron content of beryl is inversely proportional to lightness and almost directly proportional to chroma.

Iron is widely distributed in the earth's crust, and it is also an important chromogenic ion in coloured gemstones. The valence and content of iron ions and the substitution form of iron have certain influence on the colour of beryl. The w(Fe_2_O_3_) of 43 beryl samples was nondestructive measured by XRF, and the relationship between iron content and hue angle in beryl is shown in Fig. 5a. In yellow and blue beryl, the iron content is higher than that in green beryl. The highest Fe content appears in the samples with the highest hue angle 223.48 and chroma 17.00, and the lowest iron content is in the yellowish-green area, with a hue angle of 133.12. There is a significant negative correlation (Pearson's *r* = − 0.981, *R*^2^ = 0.963) between iron content and lightness shown in Fig. 5b, the higher the iron content in beryl, the lower its lightness. The chroma of beryl is almost proportional to iron content, but because of the low overall chroma value, there is little difference in chroma of beryl with different iron content.

## Conclusion

This contribution suggested Fe is the most important chromophore in yellow to light-blue beryl. The optical spectra indicated the wide absorption edge from 300 to 465 nm caused by Fe^3+^ is the origin of yellow, which does not reflect dichroism. The absorption shoulder at 650 nm in E//c-polarization is due to Fe^2+^-Fe^3+^ IVCT and leading to the blue. The characteristic absorption at 820 nm may originate from tetrahedral, octahedral or channel divalent iron and the band at 956 nm only appears in the E//c-polarized direction is attributed to spin-allowed transitions of ^VI^Fe^2+^. FTIR results show that there are few tetrahedral and octahedral substitutions, giving the explanation for the low chroma of beryl colour. The beryl colours should be divided into five groups including yellow, yellowish-green, bluish-green, greenish-blue and blue by using K-means cluster analysis. The yellow tone has much more impact on the chroma of beryl than the blue. With the help of ED-XRF, it is found that there is a certain relationship between colour and the content of iron. The yellow and blue beryl have higher iron contents than the green one. The lightness L* of beryl is negative correlated with iron content, on the contrary, the chroma C* is positive correlated with it.

## Material and methods

### Samples

A total of 95 natural beryl samples were collected, of which 13 were uncut crystals, the other 77 were cabochons, and 5 were platelets machined along the c axis and polished on both sides for spectral testing.

### Electron microprobe analysis

EMPA-1720 was used to analyze the chemical composition of beryl, The test conditions can be described as follows: acceleration voltage: 15 kV; current: 20 mA; beam spot diameter: 5 μm. The error of EMPA depends on the content of elements. When the elements is greater than 20% wt, the relative error is less than 5%, When the element content is less than 20% wt and higher than 3%wt, the relative error is less than 10%, When the element content is less than 3% wt and higher than 1% wt, the relative error is less than 30%, when the test element content is less than 1% wt, the relative error is less than 50%.

### Energy-dispersive X-ray fluorescence spectroscopy

Micro-area chemical compositions were measured using an EDX-7000 energy dispersive X-ray fluorescence spectrometer, with the following test conditions: atmosphere, oxide; a voltage of 50 kV; 108 µA. The diameter of test aperture was 3 mm.

### Fourier transform infrared spectroscopy

Fourier transform infrared spectra are tested by Tensor 27 FTIR Spectrometer produced by Bruker Company of Germany. The test conditions are as follow: scanning 32 times, the wavelength range is 4000 cm^-1^ ~ 400 cm^-1^, transmission mode, and the selected method was KBr pellets (2 mg of beryl powder mixed with 200 mg KBr).

### UV–vis spectroscopy

The UV–vis spectra are tested by using UV-3600 UV–VIS spectrophotometer. The test conditions are as follows: the range of wavelength, 300 ~ 900 nm; sampling interval, 1.0 s; single scanning mode; high scanning speed.

### Colourimetric analysis

The colours of beryl can be measured by X-Rite SP62 spectrophotometer in a standard illumination box with a 6504 K fluorescent lamp and the background is N9 gray level of Munsell neutral colour chips. In order to obtain accurate colour parameters, the value of each sample will be tested for three times. The test conditions are described as follows: refection method, not including the specular refection; observer view of 2°; measuring range, 400 ~ 700 nm; measuring time, 2.5 s; wavelength interval, 10 nm; voltage, 220 V; current, 50 ~ 60 Hz; measuring aperture, 4 mm.

## Supplementary Information

Supplementary Information.

## Data Availability

The dataset for this study is available from the corresponding author upon reasonable request.
